# The Small Auxin-Up RNA 50 *(SAUR50)* Gene from *Ammopiptanthus nanus* Negatively Regulates Drought Tolerance

**DOI:** 10.3390/plants13172512

**Published:** 2024-09-07

**Authors:** Yuanyuan Zhang, Qi Li, Mengyang Jiang, Hui Tian, Muhammad Hayder Bin Khalid, Yingge Wang, Haoqiang Yu

**Affiliations:** 1 Ecological Security and Protection Key Laboratory of Sichuan Province, College of Life Science & Biotechnology, Mianyang Normal University, Mianyang 621000, China; 2 Maize Research Institute, Sichuan Agricultural University, Chengdu 611130, China; 3 National Research Centre of Intercropping, The Islamia University of Bahawalpur, Bahawalpur 63100, Pakistan

**Keywords:** *Ammopiptanthus nanus*, auxin responsive proteins, drought stress, stomatal aperture

## Abstract

Drought stress is a primary abiotic stress that causes significant losses to forestry and agricultural production. Therefore, exploring drought-responsive genes and their regulatory mechanism is crucial for plant molecular breeding for forestry and agriculture production safety. Small auxin-up RNA (SAUR) proteins are essential in plant growth and development but show functional diversity in stress response. In this study, the transcriptome sequencing data of *Ammopiptanthus nanus* seedlings revealed that the expression of *AnSAUR50* was continuously downregulated under drought stress. Hence, the *AnSAUR50* gene was cloned and functionally analyzed in drought response. The results showed that the coding sequence of *AnSAUR50* was 315 bp in length and encoded 104 amino acids. The AnSAUR50 protein showed high conservation, possessed a SAUR-specific domain, and localized in the nucleus and cell membrane. The heterologous expression of the *AnSAUR50* gene enhanced the drought sensitivity of the transgenic *Arabidopsis* with a lower survival rate, biomass, and higher malondialdehyde content and relative electrolyte leakage. Moreover, transgenic plants showed shorter root lengths and bigger stomatal apertures, resulting in facilitating water loss under drought stress. The study indicates that *AnSAUR50* negatively regulates drought tolerance by inhibiting root growth and stomatal closure, which provides insights into the underlying function and regulatory mechanism of SAURs in plant stress response.

## 1. Introduction

Plants are frequently affected by various abiotic stresses during the growth and development process, among which drought stress is a key stress factor, causing huge losses to the world’s forestry, agricultural production, and economic development [1,2]. With climate change, population increase, unreasonable land development, and industrial development, the water demand for agriculture will double, but the availability of fresh water is predicted to drop by 50%, which seriously threatens both agriculture and humanity [3,4]. Drought changes soil permeability, leading to water loss and disrupting plant cell growth and physiological processes, thereby inhibiting plant growth and even causing plant death [5]. Therefore, it is urgent to explore new drought-related genes and the cultivation of new plant varieties with strong adaptability to unfavorable climatic conditions.

To overcome drought stress, plants undergo a series of physiological, biochemical, and molecular changes to maintain appropriate cell water potential and enzyme activity [6]. Auxin is an important phytohormone and plays a crucial role in plant growth, development, and adaptation to stress [7,8,9]. In plants, the auxin signal is mainly transduced by three families: auxin/indole-3-acetic acid (Aux/IAA), Gretchen Hagen 3 (GH3), and SAUR [10,11]. The SAUR proteins encoded by the *SAUR* genes are generally small and mostly composed of a highly conserved core domain of approximately 60 amino acid residues and diverse N-terminal and C-terminal fragments [12,13]. Numerous studies have shown that SAURs are involved in the transportation of plant auxin, the growth and development of various organs, as well as plant stress response [14,15,16,17,18]. For instance, the polar *PtSAUR8* and *Arabidopsis AtSAUR41* can improve plant drought resistance through ABA-mediated pathways, while *AtSAUR19*, peanut *AhSAUR3,* negatively regulates plant drought resistance [19,20,21,22,23], suggesting the functional diversity of *SAUR* genes in drought stress response.

*Ammopiptanthus nanus* (*A. nanus*) is an evergreen, broad-leaved, and remnant plant since the disappearance of the ancient Mediterranean in the tertiary period [24]. *A. nanus* survives in the arid deserts of central Asia, shows extreme stress-resistance ability, and can be used as a donor of excellent stress resistance gene resources. In our previous study, the *AnSAUR50* gene was found to be differentially expressed in response to drought stress (RNA-Seq data have been deposited in NCBI under BioProject accession number PRJNA684798). However, the function of *AnSAUR50* in regulating plant drought tolerance is unknown. This study aims to identify the function of *AnSAUR50* by cloning and bioinformatics analysis, subcellular localization, and overexpression analysis in transgenic *Arabidopsis thaliana* (*A. thaliana*).

## 2. Results

### 2.1. Cloning and Analysis of AnSAUR50

**Figure 1:**
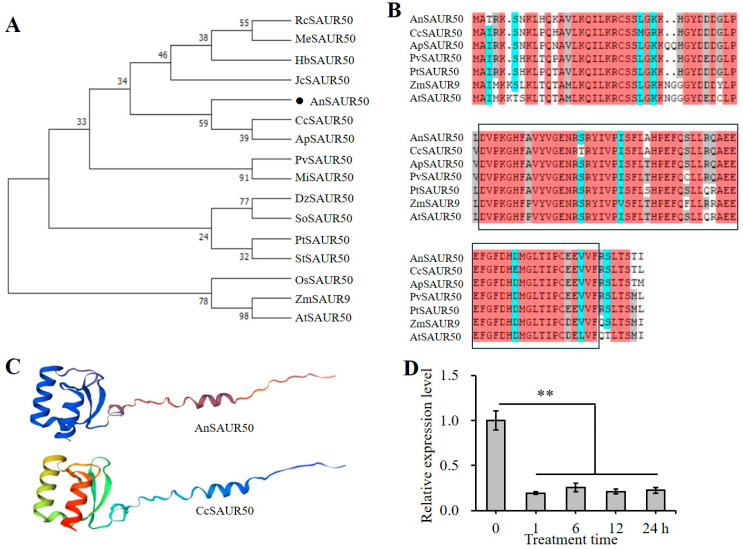
Identification of AnSAUR50. (**A**) The phylogenetic tree of AnSAUR50 and other SAURs. (**B**) Multiple alignments of AnSAUR50 and other SAURs, indicating that the SAUR-specific domain is highly conserved. Red, blue and gray backgrounds mean 100%, 75% and 50% conservation, respectively. (**C**) The predicted structure of AnSAUR50 and CcSAUR50 protein. (**D**) Expression pattern of *AnSAUR50* gene under polyethylene glycol (PEG) treatment for simulating drought conditions. The RNA-seq data were retrieved from our previous project (PRJNA684798). Box indicates the SAUR-specific domain. The black dot means AnSAUR50. ** indicates statistical significance at *p* < 0.01 level.

The *AnSAUR50* gene was cloned and showed 315 bp in length (Figure S1), encoding 104 amino acids with a molecular weight of 11.84 kDa, an isoelectric point pH of 8.62, an instability coefficient of 48.01%, and a total average hydrophobicity of −0.392, indicating it is an unstable hydrophilic protein. The phylogenetic tree analysis revealed that the AnSAUR50 protein exhibited a close relationship with the CcSAUR50 protein (GenBank: XP-020215137.1) of *Cajanus cajan* and the ApSAUR50 protein (GenBank: XP-027354820.1) of *Acacia faba*; both belonged to the same leguminous family and were classified into the same branch (Figure 1A). Meanwhile, it was found that the amino acid sequence of the AnSAUR50 protein showed high conservation and possessed a SAUR-specific domain (Figure 1B). Structural analysis revealed that there were three characteristic α-helixes and β-sheets in the AnSAUR50 protein sequence, showing a highly similar structure to the CcSAUR50 protein (Figure 1C). These results suggested that the putative sequence of AnSAUR50 cloned from *A. nanus* acted as SAUR. SAURs have been shown to regulate drought tolerance [19,20,21,22,23]. Previously, the RNA-seq of *A. nanus* was performed (PRJNA684798) and showed the downregulated expression of the *AnSAUR50* gene under drought stress (Figure 1D), suggesting the potential role of AnSAUR50 in drought stress response.

### 2.2. Subcellular Localization of AnSAUR50

**Figure 2:**
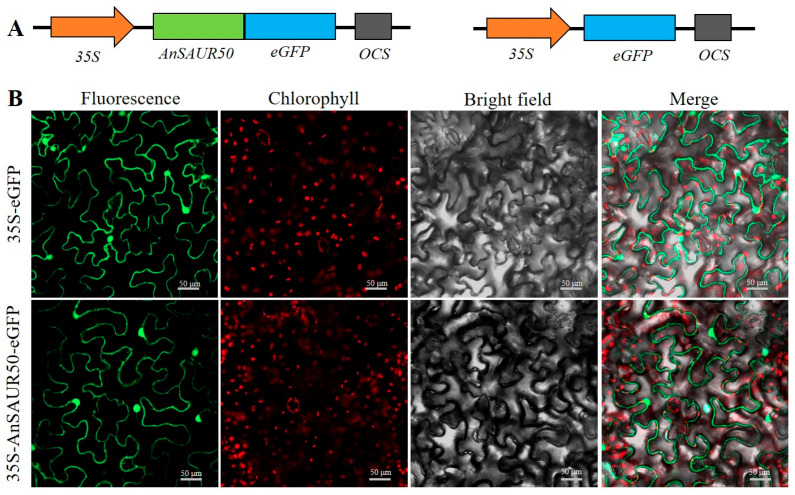
Subcellular localization of AnSAUR50. (**A**) The diagram of expression vector. *35S* promoter was used to drive *AnSAUR50*-*eGFP*, and *eGFP* gene. *OCS*, the 3’-flanking region of octopine synthase gene, was used as terminator. (**B**) The photos of eGFP fluorescence signal in the cells transformed by *35S*-*AnSAUR50*-*eGFP* and *35S*-*eGFP*. Scar bar was 50 μm.

To detect the subcellular localization of the AnSAUR50 protein, the *35S*-*AnSAUR50*-*eGFP* vector was constructed (Figure 2A) and transformed into tobacco leaf cells for transient expression. As shown in Figure 2B, the green fluorescence signal of eGFP in the cells transformed by *35S*-*AnSAUR50*-*eGFP* was observed in the whole cells, including the nucleus and cytoplasm, which was similar to the leaf cells transformed by the control *35S*-*eGFP* vector. This indicated that the AnSAUR50 protein was localized in the nucleus and cytoplasm. 

### 2.3. Identification of Transgenic Arabidopsis Lines

**Figure 3:**
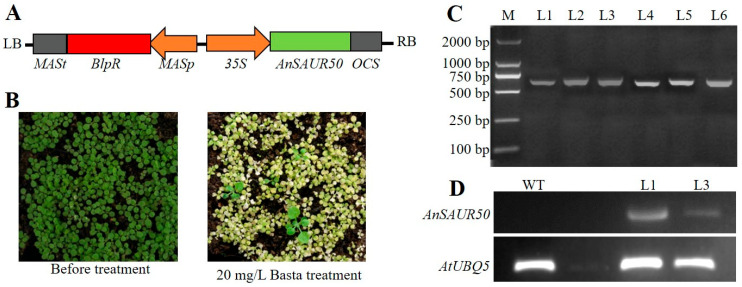
Identification of transgenic lines. (**A**) The diagram of expression vectors. The *35S* and *OCS* sequences were used to drive and terminate *AnSAUR50* gene. *MASp* and *MASt* mean promoter and terminator of mannopine synthase. *BlpR*, encoding phosphinothricin acetyltransferase confers resistance to bialophos or phosphinothricin. (**B**) Phenotype of basta screening for transgenic plants. (**C**) PCR detection of transgenic lines. M, DNA marker DL2000. L1 to L6 were T_1_ transgenic lines overexpressing *AnSAUR50*. (**D**) RT-PCR analysis. The *AtUBQ5* was used as a reference gene. WT, wild type. L1 and L3 were homozygous lines expressing *AnSAUR50* gene.

To explore the function of AnSAUR50 in drought response, the AnSAUR50 overexpression vector (Figure 3A) was stably transformed into Arabidopsis using the flower-dipping method [25]. Six independently positive T_1_ lines were screened by Basta (Figure 3B) and PCR detection (Figure 3C) and used to generate T_3_ generation. In the homozygous T_3_ generation, the AnSAUR50 sequences were amplified from the cDNA of lines 1 and 3, but not amplified in wild type (WT) through RT-PCR. The reference gene of AtUBQ5 was amplified in both T_3_ generation lines and WT control, confirming the success of the transgenic Arabidopsis lines (Figure 3D).

### 2.4. Expression of AnSAUR50 Inhibited Drought Tolerance of Transgenic Arabidopssis

**Figure 4:**
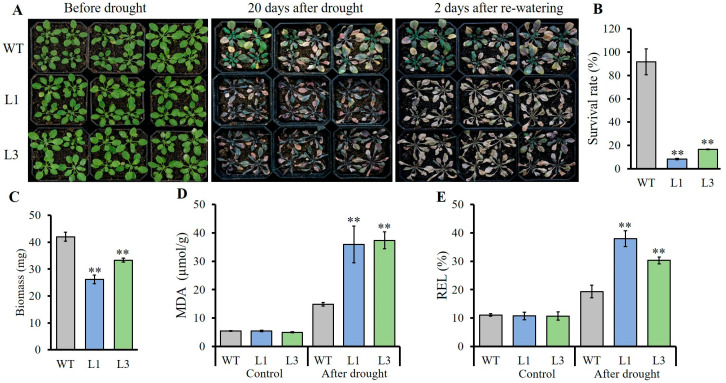
Overexpression of *AnSAUR50* enhanced drought sensitivity of transgenic *Arabidopsis*. (**A**) Phenotype of *A. thaliana* transformed by *AnSAUR50* under drought stress. (**B**) Survival rate of each line after drought stress. (**C**) Biomass of every line after drought stress. (**D**) Malondialdehyde (MDA) content. (**E**). Relative electrolyte leakage (REL). WT, wild type. L1 and L3 were homozygous lines expressing the *AnSAUR50* gene. ** indicates statistical significance at *p* < 0.01 level.

The downregulated expression of *AnSAUR50* gene by drought stress suggested its potential role in regulating drought tolerance; hence, all plants were grown in soil and used for drought treatment. As shown in Figure 4, when the 3-week-old seedlings with the same size of each line were subjected to without watering for 20 days, overexpressing lines L1, L3, and WT both showed the wilting phenotype. The L1 and L3 leaves were significantly more severely wilting than those of the WT, whereas some WT leaves were still green and stretched (Figure 4A). After 2 days of rewatering, the survival rates of L1 and L3 were only 8.3% and 16.7%, respectively, which were significantly lower than those of WT with a 91.7% survival rate (Figure 4B). Likewise, it was found that the biomasses of L1 and L3 were 26.2 and 33.3 mg, respectively, which were significantly lower than WT of 42.0 mg (Figure 4C). Meanwhile, the malondialdehyde (MDA) content and relative electrolyte leakage (REL) of all lines were evaluated. Under normal conditions, the MDA content and REL were kept low and showed no significant difference among the three lines. In contrast, the MDA content and REL of each line were dramatically increased after drought treatment. Moreover, compared with WT, the MDA content and REL of L1 and L3 lines were significantly higher (Figure 4D,E). These results suggested that the overexpression of the *AnSAUR50* gene decreased the drought tolerance of transgenic *Arabidopsis*.

### 2.5. Expression of AnSAUR50 Inhibited Root Length of Transgenic Lines under Drought Stress

**Figure 5:**
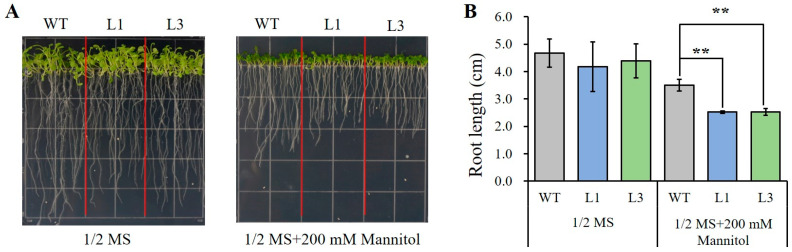
Overexpression of *AnSAUR50* resulted in root growth retardation of transgenic *Arabidopsis* under simulated drought conditions. (**A**) Root phenotype. (**B**) The statistics of root length. WT, wild type. L1 and L3 were homozygous lines expressing *AnSAUR50* gene. ** indicates statistical significance at *p* < 0.01 level.

When seeds of all transgenic lines and WT were sown on 1/2 MS medium for 14 days, the growth status of transgenic lines was basically consistent with that of the control and showed no significant difference in root length among different lines. However, after 14 days of cultivation on 1/2 MS medium with 200 mM mannitol, although the growth of each line was inhibited, the L1 and L3 lines exhibited shorter primary roots than that of the WT (Figure 5A,B), indicating that expression of *AnSAUR50* inhibited root growth of transformed lines under simulated drought stress.

### 2.6. Expression of AnSAUR50 Facilitated Stomatal Aperture of Transgenic Arabidopsis under Drought Stress

**Figure 6:**
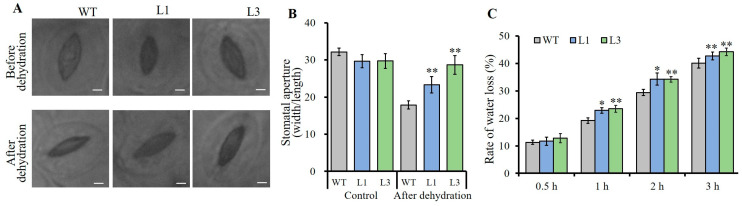
Overexpression of *AnSAUR50* altered stomatal aperture of transgenic *Arabidopsis*. (**A**) Phenotype of stoma of each line. The leaf samples of every line were excised for 90 min of dehydration and used to detect stomatal aperture. Scar bar = 20 µm. (**B**) The statistical data of stomatal aperture. (**C**) Water loss rate of detached leaves. WT, wild type. L1 and L3 were homozygous lines expressing *AnSAUR50* gene. * and ** indicate statistical significance at *p* < 0.05 and *p* < 0.01 level.

To further dissect the clues causing the alteration of drought tolerance of *AnSAUR50*-overexpressing lines, the stomas of all lines were monitored. The results exhibited that the stomas were kept open and showed higher stomatal aperture and no significant difference among all lines before dehydration (Figure 6A). However, when the leaves were detached for dehydration of 90 min, some stomas were closed, and the stomatal apertures of L1, L3 lines, and WT were decreased. Among them, the stomatal apertures of the L1 and L3 lines were still bigger than the WT (Figure 6A,B). Meanwhile, the results of the water loss rate of detached leaves found that L1 and L3 lines maintained a higher water loss rate than that of WT at 1, 2, and 3 h of dehydration (Figure 6C).

## 3. Discussion

The *SAUR* gene was first discovered in soybean hypocotyls and was found to be a transcript induced by auxin [26]. Subsequently, the *SAUR* genes were discovered in various plant species and encoded a kind of unique auxin-responsive factor SAUR family in plants [27,28,29,30,31]. In this study, the amino acid sequence of AnSAUR50 showed high conservation and contained a highly conserved SAUR-specific domain with 60 aa (Figure 1), which was consistent with other SAUR50s [31,32]. Likewise, AnSAUR50 exhibited a similar structure to CcSAUR50 and was located in a subclade with CcSAUR50 (Figure 1), suggesting that the sequence of AnSAUR50 cloned from *A. nanus* functioned as SAUR.

Previous studies have proved that the subcellular localization of SAUR proteins showed diversity. Our study confirmed that the AnSAUR50 protein was simultaneously localized in the nucleus and cytoplasm (Figure 2), which was consistent with the subcellular localization of AtSAUR32 and TaSAUR75 [33,34]. However, it has been reported that AtSAUR41 localized to the cytoplasm, while AtSAUR19 and AtSAUR63 were localized mainly in the plasma membrane [35,36]. The diversity of subcellular localization may be related to their functional diversity in plant growth, development, and stress response.

Gene expression pattern reveals its potential function. Here, the *AnSAUR50* gene of *A. nanus* was continuously downregulated under drought (Figure 1), indicating that *AnSAUR50* was involved in the response to drought. Subsequently, the *AnSAUR50* gene was overexpressed in *Arabidopsis*. The results of phenotyping revealed that overexpressing plants with *AnSAUR50* exhibited more drought sensitivity than WT with lower survival rate, biomass, as well as higher MDA content and REL (Figure 4), confirming that AnSAUR50 negatively regulated drought tolerance. Available studies have shown that roots can respond to moisture and coordinate responses to drought because the root is the first defensive line to environmental stimuli from soil [3,37,38]. The enhancement of drought sensitivity of transgenic plants could be explained by the inhibition of root growth and stomatal closure in transgenic plants overexpressing the *AnSAUR50* gene (Figure 5 and Figure 6). Meanwhile, the opening of the stoma in transgenic lines facilitated water loss and the wilting of leaves (Figure 6). Many studies have shown that overexpression of SAUR genes can alter drought resistance in transgenic plants [19,33,34,35,36]. The *AtSAUR41* could regulate root meristem patterning and increase primary root length and the number of lateral roots in overexpressing *Arabidopsis* [20,36]. Overexpression of the *TaSAUR75* gene facilitated root length to enhance the salt and drought tolerance of *Arabidopsis* [33]. Overexpression of *AtSAUR19* and *AtSAUR63* also exhibited larger stomatal apertures and accelerated water loss compared to WT, resulting in accelerated leaf water transpiration and increased sensitivity to drought [21,22,39]. AtSAUR32 interacted with clade-A PP2C proteins (AtHAI1 and AtAIP1) to regulate drought sensitivity via ABA, signaling in *Arabidopsis* [34]. The *AhSAUR3* gene from *Arachis hypogaea* likewise negatively regulated drought tolerance in transgenic *Arabidopsis* [23]. The *SAUR* gene family has numerous members and diverse functions, especially in response to abiotic stressors such as drought stress. Taken together, the study provides insights into further underlying the mechanism of SAUR genes in regulating the drought stress response.

## 4. Materials and Methods

### 4.1. Cloning and Bioinformatics Analysis of AnSAUR50

According to our previous study, the total RNA of *A.nanus* was extracted using a RNA extraction kit (KaKaRa, Kusatsu, Japan) and used to synthesize cDNA using a reverse transcription kit (KaKaRa, Kusatsu, Japan) [40]. The specific primers AnSAUR50-F/AnSAUR50-R (Table S1) were designed, synthesized, and used for amplifying the open reading frame (ORF) of *AnSAUR50* from a cDNA sample using the high-fidelity enzyme Phanta max (Novozan, Nanjing, China). The PCR products were sent for sequencing at Sangon Biotech (Shanghai, China). After sequencing, the sequence was analyzed and used to translate the protein sequence, which was used as a probe to search for homologous proteins from the NCBI protein database (https://www.ncbi.nlm.nih.gov/guide/proteins/. accessed on 17 May 2022). The AnSAUR50 amino acid sequence was aligned with those homologous proteins using Cluster X2.1 software (http://mac.softpedia.com/get/Math-Scientific/ClustalX.shtml. accessed on 17 May 2022), and an evolutionary tree was constructed using the molecular evolutionary tree analysis tool MEGA7.2 (https://mega.software.informer.com/7.2/. accessed on 17 May 2022). The conserved domain and structure of the candidate protein was analyzed using the CDD database (https://www.ncbi.nlm.nih.gov/Structure/cdd/cdd.shtml. accessed on 27 October 2022) and (https://swissmodel.expasy.org/. accessed on 27 October 2022), respectively. The data of RNA-seq of *A. nanus* under simulated drought stress with 20% PEG6000 (NCBI BioProject number PRJNA684798) were used to analyze the expression level of *AnSAUR50*.

### 4.2. Identification of Subcellular Localization of AnSAUR50

The primer pairs AnSG-F and AnSG-R (Table S1) were designed using CE Design V1.04 software and used to amplify the ORF of *AnSAUR50* without a stop codon. The PCR products were constructed into the *Xba* I and *Spe* I sites of the pCAMBIA2300-*35S*-*eGFP* plasmid to generate the *35S*-*AnSAUR50*-*eGFP*, which was used for transient expression. As described by Lu et al. [41], the *35S*-*AnSAUR50*-*eGFP* and *35S*-*eGFP* empty plasmid was transformed into *Agrobacterium GV3101* and then used to inject the *Nicotiana benthamiana* leaves for 48 h at room temperature. The eGFP and chloroplast spontaneous fluorescence was observed and photographed at excitation wavelengths of 488 and 580 nm under the LSM800 confocal laser microscope (ZIESS, Oberkochen, Germany), respectively.

### 4.3. Transformation and Screening of Arabidopsis thaliana

The pEarleyGate 100-*AnSAUR50* vector was constructed using pENTR™/D-TOPO^TM^ Cloning Kits (Invitrogen, Carlsbad, CA, USA) and a Gateway^TM^ LR Close™ Enzyme Mix Kit (Invitrogen, Carlsbad, CA, USA). The successfully constructed vector was transferred into *Agrobacterium GV3101*, and the soaking of flower buds was repeated three times using the Arabidopsis floral dip method to transfer the gene into *A. thaliana*. 

The seeds of T_0_ generation were vernalized at 4 °C for 2 days and evenly sown in nutrient soil (nutrient soil–vermiculite = 3:1) at 25 °C 10 h/20 °C 14 h with a relative humidity of 60–70%, and light intensity of 200 μmol·m^−2^·s^−1^ for 14 h/dark 10 h. The two-week-old seedlings were sprayed with 20 mg/L basta three times within two days to screen positive transgenic plants. The survival plants were cultivated under the same conditions to produce the next generation until homozygous T_3_ generation was screened and used in the next study.

### 4.4. Identification of Target Genes by PCR and RT-PCR

The total DNA and RNA of each line were extracted using the CTAB method and RNA extraction kit (KaKaRa, Kusatsu, Japan), respectively. The total RNA was used for reverse transcription to synthesize cDNA using PrimeScript^TM^ 1st Strand cDNA Synthesis (TaKaRa, Kusatsu, Japan). The universal primers attR-F/attR-R (Table S1) were used to perform PCR detection. The primers AnSAUR-F/AnSAUR-R (Table S1) were designed, synthesized, and used to detect the expression of *AnSAUR50* in transgenic lines using RT-PCR. The primer pair AtUBQ-F and AtUBQ-R (Table S1) was used to amplify the polyubiquitin (UBQ) family *AtUBQ5* gene and used as an internal reference gene. The amplification products were separated by 1.5% agarose gel electrophoresis to confirm the expression of *AnSAUR50*.

### 4.5. Phenotyping

The seeds of homozygousT_3_ generation and WT were sown into nutrient soil (nutrient soil–vermiculite = 3:1) and cultivated under 20–22 °C, 10 h light/14 h darkness, and relative humidity of 60–70%. According to our previous study, the one-month-old seedlings were subjected to natural drought treatment without watering [42]. About 20 days later, photos were taken and recorded when the drought-treated plants showed symptoms of wilting. Subsequently, all plants were treated by re-watering, recovered for 2 days, and photographed. Meanwhile, the survival rate of each line was monitored. The aboveground parts of each plant were sampled, dried at 80 °C for 24 h to constant weight, and used to measure biomass.

According to the methods described by Lv et al. [43] with minor modifications, the MDA content and REL were detected. In brief, about 0.3 g leaves of transgenic lines and WT treated with drought were sampled and used to test absorbance values at 450, 532, and 600 nm using a spectrometer. The concentration of MDA was calculated using the following formula: C (μmol/L) = (6.45 × (A_532_ − A_600_) − 0.65 × A_450_) × 2 × V)/W, where V and W refer to the volume of 10% trichloroacetic acid in the reaction system and the weight of samples, respectively. The REL of each line was measured as described by Liu et al. [44] with some modifications. The leaves of the transgenic line and WT were sampled and submerged in 20 mL of distilled water followed by incubation in boiling water (100 °C) for 20 min; the conductivities EL1 (initial before heating) and EL2 (after cooling) were measured using a conductivity meter (Model DDS-308+, Shanghai, China). The REL was calculated as (EL1/EL2) × 100%.

### 4.6. Measurement of Root Length

Root growth assay was conducted according to the methods described by Guo et al. [33]. The seeds of overexpression lines and WT were surface-sterilized, germinated on 1/2 MS plates (control) or supplemented with 200 mmol/L mannitol, and cultured in a greenhouse under 25 °C 10 h/20 °C 14 h with relative humidity of 60–70%, and light intensity of 200 μmol·m^−2^·s^−1^ under 14 h/dark 10 h. Three weeks later, the growth status and primary root length of each line were monitored.

### 4.7. Measurement of Stomatal Aperture and Water Loss

The stomatal aperture of epidermal peels was assessed according to Zhang et al. [45] with minor modifications. Leaves from 4-week-old plants were detached and incubated for 90 min at 25 °C under white light (150 pmol·m^−2^·s^−1^). Gelatin was applied on the back of the leaf cover and the epidermis was carefully peeled off. The morphology of stomas was observed under the LSM800 confocal laser microscope (ZIESS, Carlsbad, CA, USA). The length and width of stomas were measured using the ImageJ tool and used to calculate the stomatal aperture. The water loss assay was assessed in detached leaves of 4-week-old transgenic lines and WT plants placed in a growth chamber with 40% relative humidity. As described by Yoo et al. [46], the fresh weight (FW) was recorded immediately when the leaves were detached. Then, the FW was weighted at the interval times 0.5, 1, 2, and 3 h, respectively, and defined final FW. The proportion of water lost was calculated as (Initial FW − Final FW)/Initial FW × 100%.

### 4.8. Data Analysis

All experiments were performed with three replicates. The data were shown as mean values ± standard error (SE). Statistical analysis of the data was performed using Microsoft Excel and IBM SPSS Statistics 20. Asterisk symbols (* *p* < 0.05; ** *p* < 0.01) indicate a significant difference in comparison to WT. 

## 5. Conclusions

The *AnSAUR50* gene was 315 bp in length, encoding 104 amino acids. The AnSAUR50 protein showed high conservation and localized in the nucleus and cytoplasm. The expression of *AnSAUR50* was continuously downregulated in *A. nanus* under drought stress. Overexpression of AnSAUR50 negatively regulated drought tolerance in transgenic *Arabidopsis* via inhibiting root growth and stomatal closure. The study provides insights into the underlying function and regulatory mechanism of SAURs in plant stress response.

## Data Availability

Data supporting the findings of this study are available in the article and its Supplementary Materials.

## Supplementary Materials

The following supporting information can be downloaded at: https://www.mdpi.com/article/10.3390/plants13172512/s1, Figure S1: Cloning and sequencing of *AnSAUR50*. (A) Agarose gel assay of AnSAUR50. (B) Alignment of *AnSAUR50* sequencing; Table S1: Primers used in this study.
