# Supplementary material for: The Small Auxin-Up RNA 50 (SAUR50) Gene from Ammopiptanthus nanus Negatively Regulates Drought Tolerance
Source: Plants (Basel). 2024 Sep 7;13(17):2512. doi: 10.3390/plants13172512 (PMC11397199; doi:10.3390/plants13172512)
Supplement: Supplementary file 1 [file plants-13-02512-s001.zip › plants-3131376-supplementary.pdf]

Table S1 Primers used in this study

Primer	Sequence
AnSAUR50-F	5'-ATGGCCACTAGAAAATCAAACAAGC-3'
An SAUR50-R	5'-TCATCTGATCGTTGATGTTAGAGAGCG-3'
AnSG-F	5'-**CAGGGTACCCGGGGATCC***TCTAGA* ATGGCCACTAGAAAATCAAACAAGC-3'
AnSG-R	5'-**CGCCCTTGCTCACCATGGT***ACTAGT* ACTAGTACCATGGTGAGCAAGGGCG-3'
AtUBQ-F	5'-CTAACGGGGAAGACCATAACC-3'
AtUBQ-R	5'-CTTAACCTTCTTATGCTTGTG-3'
attR-F	5'-TACTGTAAAACACAACATATCCAGTC-3'
attR-R	5'-AAAACACAACATATCCAGTCACTATG-3'

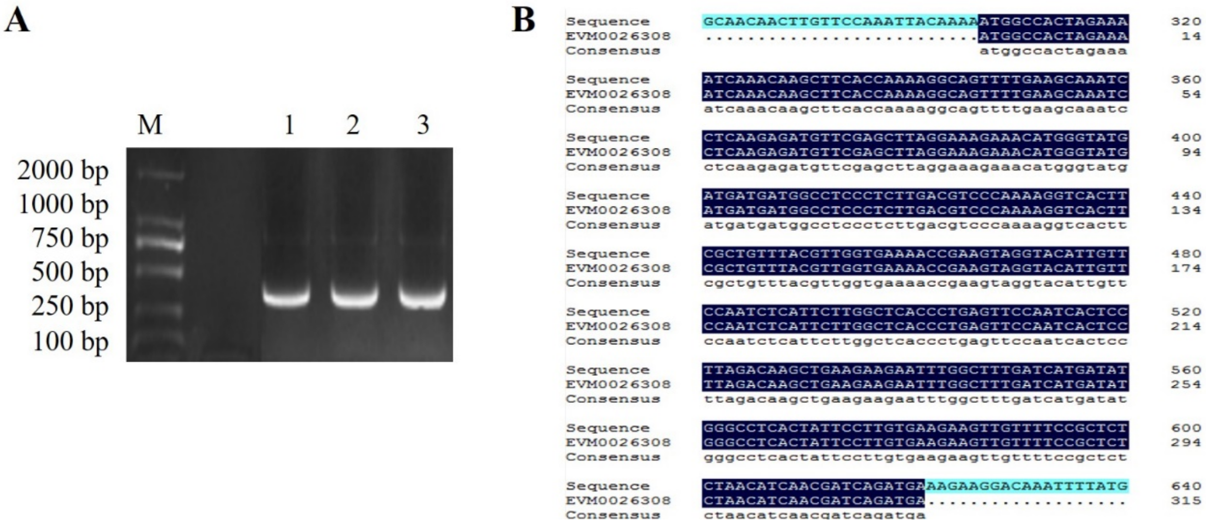

Figiure. S1 Cloning and sequencing of *AnSAUR50*. (**A**) Agarose gel assay of *AnSAUR50*. (**B**) Alignment of *AnSAUR50* sequencing.
